# The protective effects of Nicorandil on renal function in patients undergoing coronary interventions: a systematic review and meta-analysis

**DOI:** 10.1186/s12882-025-04564-8

**Published:** 2025-12-01

**Authors:** Ahmed A. Abo Elnaga, Ibrahim Saleh Alawadi, Abdelrahman M. Elettreby, Mohammed Ashry, Hebatalla Ossama, Hazem A. Farouk, Alaa O. Hussein, Rehab Refaat, Leina Sherif, Emad Samaan

**Affiliations:** 1 Faculty of Medicine, Mansoura University, Mansoura, Egypt; 2 Internal Medicine and Nephrology, Faculty of Medicine, Mansoura University, Mansoura, Egypt; 3 Mansoura Manchester Research Society, Mansoura, Egypt

**Keywords:** Contrast-induced nephropathy, Coronary intervention, Kidney disease, Nicorandil

## Abstract

**Background:**

Contrast-induced nephropathy (CIN) is a serious complication in patients undergoing cardiac interventions, especially in patients with chronic kidney disease. This study aims to assess the efficacy of Nicorandil in CIN reduction and its impact on renal function outcomes.

**Methods:**

A comprehensive search was conducted from inception to September, 2024 across four databases. Randomized controlled trials (RCTs) assessing the efficacy of Nicorandil for patients undergoing coronary procedures were included.

**Results:**

Based on analysis of 11 clinical trials involving 2837 patients, Nicorandil significantly reduced the incidence of CIN compared to control group (RR = 0.37, 95%CI [0.27 to 0.49], *P* < 0.001) without heterogeneity. Nicorandil group was superior to control group in reducing the rise in serum creatinine level at 24, 48 and 72 h (MD= -4.45 umol/L, 95%CI [-7.94 to -0.97], *P* = 0.01), (MD= -5.57 umol/L, 95%CI [-8.95 to -2.20], *P* < 0.001) and (MD= -5.70 umol/L, 95%CI [-9.57 to -1.82], *P* = 0.004) respectively. However, there was no statistically significant difference between Nicorandil group and control group regarding estimated glomerular filtration rate (eGFR) measured at 24, 48 and 72 h (MD = 2.17, 95%CI [-2.03 to 6.37], *P* = 0.31), (MD = 1.89, 95%CI [-0.15 to 3.93], *P* = 0.17) and (MD = 2.26, 95%CI [-0.37 to 4.89], *P* = 0.09) respectively. Nicorandil showed no statistically significant difference between both groups regarding any major adverse event, including stroke, myocardial infarction, conversion to emergency Percutaneous Coronary Intervention (PCI), or urgent need for dialysis.

**Conclusion:**

Nicorandil had an effective role in reducing the incidence of CIN, lower rise in creatinine, and a good safety profile in patients undergoing coronary interventions.

**Clinical trial number:**

Not applicable.

**Supplementary Information:**

The online version contains supplementary material available at 10.1186/s12882-025-04564-8.

. 

## Introduction

Percutaneous coronary intervention (PCI) and Coronary angiography (CAG) are common interventional procedures for treatment and diagnosis of coronary heart diseases, which have increased steadily in routine medical practice in recent years. However, the application of contrast agents for patients undergoing these procedures usually carries a high risk for acute kidney injury (AKI), especially in patients with poor kidney function [1, 2]. Patients with chronic kidney disease (CKD) undergoing PCI or CAG are associated with a high risk of contrast-related nephropathy and have a 3-fold increased risk of mortality compared to those with normal renal function parameters [3, 4].

Contrast-induced nephropathy (CIN) is characterized by worsening of renal function, typically occurring within 5 days after intravenous contrast administration which occurs in 3 to 14% of patients undergoing PCI or CAG. The incidence is even higher in patients with renal impairment and coexisting comorbidities and ranks as the third common cause for hospital-acquired acute renal injury [5–7]. The specific underlying mechanisms of CIN have not been fully clear. They are attributed to the application of contrast agents, which cause damage to the renal and vascular endothelium systems either directly or indirectly through the induction of hypoxia, reactive oxygen species, or endothelin mediators with subsequent decline in estimated glomerular filtration rate (eGFR) and kidney injury [8, 9].

Currently, there is no specific or definitive treatment for CIN and all guidelines and recommendations focus on prevention and prophylactic measures. The recent guidelines recommend the application of perioperative intravenous saline hydration to prevent CIN among patients with CKD [10–13]. However, the efficacy of hydration therapy is controversial as a recent clinical trial with a large sample size by Nijssen et al. in 2017 found that non-hydration therapy was non-inferior to hydration therapy regarding CIN reduction. On the other hand, a study by Liu et al. in 2019 reported a significant reduction in CIN in hydration groups. Furthermore, hydration therapy is time-consuming and associated with high costs [14–16]. Therefore, many studies have been conducted to explore additional drugs for preventing the occurrence of CIN, such as N-acetylcysteine, statins, but results were debatable [17–19].

Nicorandil, a nicotinamide nitrate, is a hybrid compound derived from an adenosine triphosphate-sensitive potassium channel (K-ATP channel) opener and a nitric oxide donor, and has been widely used in the treatment of angina pectoris and acute heart [20, 21]. Additionally, it ameliorates ischemia-reperfusion injury in the kidney due to its antioxidant properties as reported in preclinical studies [22].

Pranata et al. evaluated the efficacy of Nicorandil in reducing the incidence of CIN in patients with poor kidney function undergoing cardiac interventions and they found that Nicorandil is associated with a lower risk of CIN [23]. Despite these insights provided by literature and previous meta-analysis, there is still a gap of knowledge regarding changes in renal function parameters, like changes in creatinine and glomerular filtration rate and possible adverse events. So, our systematic review and meta-analysis of randomized clinical trials (RCTs) aim to provide a comprehensive updated analysis for the efficacy of Nicorandil and associated potential adverse events in patients undergoing elective PCI or CAG with renal dysfunction, with assessment of certainty of evidence.

## Methods

### Protocol and registration

We conducted this systematic review and meta-analysis according to the Preferred Reporting Items for Systematic Reviews and Meta-Analyses (PRISMA) criteria [24], as well as specific instructions from the Cochrane Handbook for Systematic Reviews of Interventions [25], as shown in Table S1 in supplementary file. Our protocol was registered at Prospero with the number (CRD42024584946).

### Eligibility criteria

#### Inclusion criteria

We included studies in our review if they satisfied the following criteria:

1. * Population*: patients with poor kidney function with impaired eGFR or creatinine clearance below 60 mL/min undergoing cardiac interventions (CAG, PCI, or cardiac catheterization).
2. * Intervention*: intravenous and oral Nicorandil in different doses additional to hydration.
3. * Comparator*: hydration using normal saline (control group).
4. * Outcome*:

(i) ** Primary outcomes**: Number of patients experiencing CIN.
(ii) ** Secondary outcomes**: Change in Serum creatinine level, change in eGFR, Change in Serum Cystatin C level, change in blood urea nitrogen (BUN), and adverse events, such as Heart failure, cardiac death, myocardial infarction, stroke, and patients who underwent temporary dialysis).

(5) Study design: RCTs.

#### Exclusion criteria

We excluded studies that do not meet the established inclusion criteria for this study. Observational studies, case reports, case series, non-randomized controlled trials, and studies that assess interventions or comparators unrelated to the research question were excluded.

### Search strategy

We searched for eligible articles on Cochrane Central Register of Controlled Trials, PubMed, Scopus and Web of Science from inception up to September 15, 2024. The following keywords were searched using the Boolean operators (Nicorandil and ((contrast induced nephropathy) OR (Contrast-induced nephropathy) OR (contrast related nephropathy) OR nephropathy OR (kidney disease*)) without additional filters or time restrictions. The detailed search strategy is shown in the supplementary file.

### Study selection and data extraction

After retrieving citations from electronic databases, the records were imported directly into Rayyan software [26]. Three authors independently screened the articles by reviewing their titles and abstracts and any conflict was solved by a senior author. Then, full-text screening was conducted based on the eligibility criteria of our study, followed by the data extraction from articles that met the eligibility criteria. Four authors independently extracted all possible data from the included studies into Microsoft excel sheets. This Excel sheet included the study ID, study design, study location, definition of CIN by each study, total sample size and sample size of each group, age, sex, body mass index (BMI), in addition to the dose and method of intervention, and baseline serum level of creatinine, contrast volume, drug intake, and associated medical conditions.

### Risk of bias assessment and certainty of evidence

Three authors blindly performed the quality assessment of the included studies using the risk of bias tool for randomized trials 2 (RoB 2) [27], which involves five domains evaluated by a set of questions, including allocation and randomization processes, deviations from intended interventions, missing outcome data, variations in outcome measurements, and selection of reported results. Each study was rated to have one of three levels of bias: low risk, some concerns, or high risk and disagreements were discussed by a senior author. Additionally, we assessed the quality of evidence using the Grading of Recommendations Assessment, Development and Evaluation (GRADE) approach to evaluate certainty levels [28].

### Statistical analysis and heterogeneity

Meta-analysis was performed using Review Manager software (RevMan v.5.4) [29] and STATA v.17 [30]. We used mean difference (MD) to pool continuous outcomes with a 95% confidence interval (CI) and risk ratio for binary outcome data. A significance threshold of *p* < 0.05 was chosen. If means and standard deviations were not directly reported, we derived them from other statistical measures. We extracted the change from baseline values instead of post treatment values. In case of the absence of the SD of change, an estimate was computed using an assumed correlation coefficient of 0.8 estimated from one of the included studies (KO 2013) [31]. We utilized the Plot Digitizer online app to extract data presented in figures [32]. If the results were presented in various units, we opted for one unit and standardized the others accordingly using an online tool [33]. To address heterogeneity between studies and obtain a more conservative effect estimate, we employed a random-effects model [34]. We assessed the presence and extent of heterogeneity using the Chi-square and I-square tests. I-square values were interpreted as follows: 0–50% indicated mild heterogeneity, 50–70% was considered as moderate heterogeneity, and values above 50% were deemed significant. Heterogeneity was considered statistically significant if the Chi-square test’s alpha level was below 0.1. Robustness was assessed via leave-one-out sensitivity analyses for all outcomes, with the pooled estimate recalculated after removing each study. We conducted leave-one-out analysis on all outcomes to evaluate the robustness of the results. Additionally, a subgroup analysis was performed based on various time points for the endpoint. Publication bias was assessed visually and by statistical tests [35, 36]. Finally, we conducted an ad hoc meta-regression, using age, BMI, and contrast volume as independent predictors.

## Results

### Study selection

**Fig. 1:**
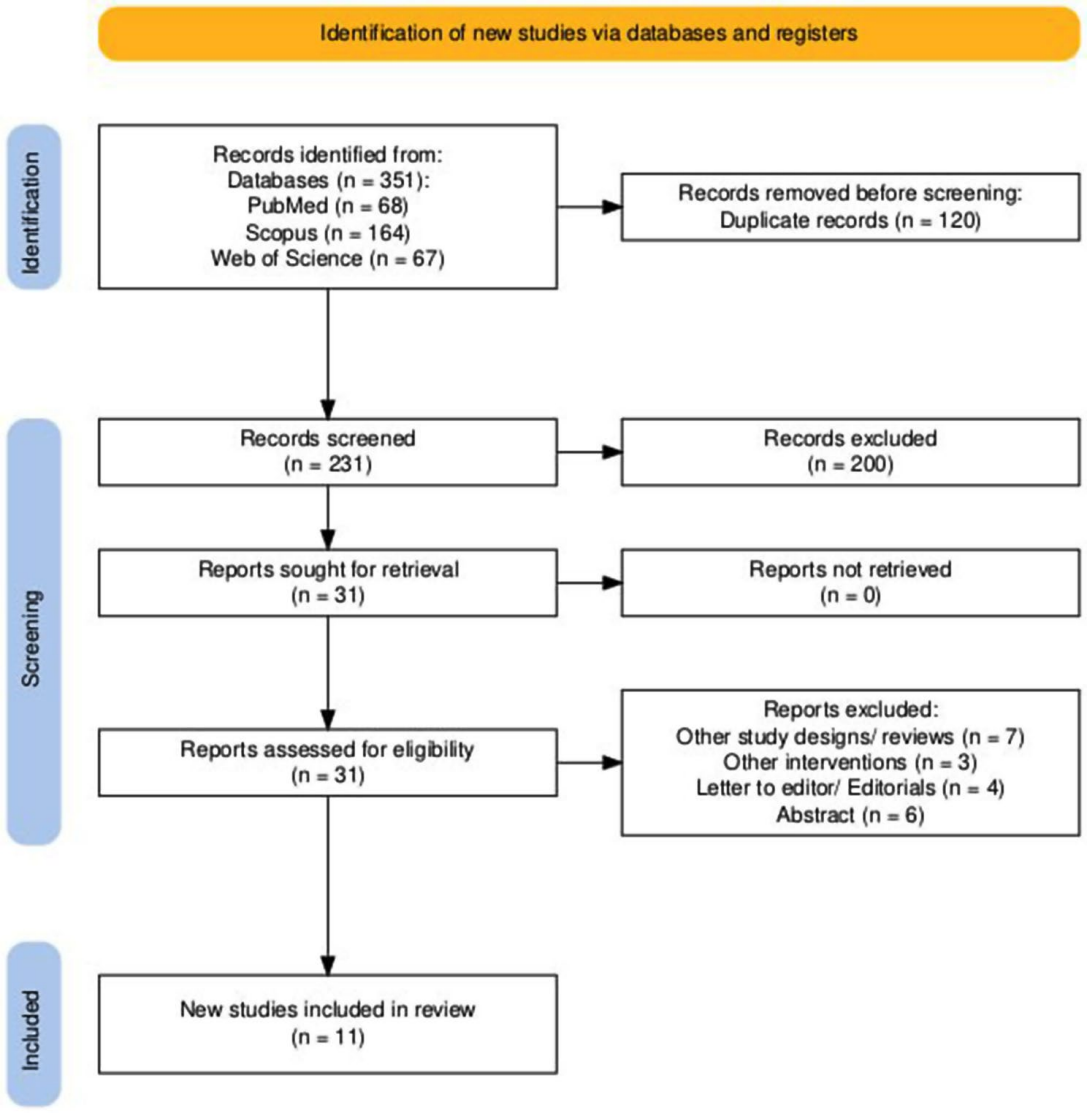
PRISMA flow diagram for study selection

The initial literature search was undertaken across PubMed, Scopus, Web of Science, and Cochrane, and retrieved a total of 351 relevant articles. A total of 120 records were removed due to duplication. After rigorous screening of the 231 remaining articles based on the eligibility criteria, 31 articles were included from title and abstract screening. Finally, eleven articles were eligible for analysis following a meticulous examination of their full text after the exclusion of twenty records for various reasons. PRISMA flow diagram representing the selection process is illustrated in Fig. 1.

### Study characteristics

Eleven prospective RCTs [20, 31, 37–45] involving a total of 2837 patients, published between 2013 and 2023 and conducted on adult patients undergoing elective cardiac procedures (PCI, CAG or cardiac catheterization) at high risk for CIN or with renal dysfunction defined as (eGFR below 60 ml/min/1.73m2, serum creatinine higher than 132.6 mol/L or creatinine clearance below 60 mL/min) in all included studies except Yusuf 2023 which included patients with eGFR between 30 and 89 mL/min/1.73 m2. All included studies were conducted in different medical centers in Asian countries, including China, Iran, India, Korea and. Furthermore, most of included studies were randomized, open-label parallel-group trials except in Kebar2023, Fan 2016, and Zeng 2019, which were double-blinded trials. The CIN was defined as an increase in serum creatinine ≥ 25% and/or ≥ 0.5 mg/day within 72 h after exposure to the contrast medium in five studies, while it was defined as the same increase within 48 h in four studies. Kebar 2023 and Yusuf 2023 considered ≥ 0.3 mg/dL increase in serum creatinine from baseline within 48 h to 72 h to define CIN according to updated guidelines.

The Intervention was oral and intravenous Nicorandil within 30 min to 3 days before the cardiac procedure and continued for up to 3 days after the procedure, in addition to hydration using intravenous normal saline. All control groups received intravenous hydration as a study comparator. The summary of included studies and characteristics of participants at baseline are shown in Tables 1 and 2.

**Table 1:** Summary of the included studies

Study ID	Study design	Location	Target population /Inclusion criteria	Exclusion criteria	Total sample size	Intervention	*n*	Comparator	*n*	Def. of CIN	Type of intervention	Outcome addressing
Kebar et al., 2023	RCT	Iran	patients, aged between 18 and 75 yearswith chronic kidney disease with glomerular filtration rate (GFR) below 60 mL/min/ 1.73m2, and candidates for coronary angiography with cardiac ejection fractional (EF) more than 40%	patients with symptoms of hypotension (systolic pressure < 90 mmHg or diastolic pressure < 50 mmHg), patients with a history of allergic reactions to contrast agents, and patients requiring emergency angiography	279	G1: oral nicorandil tablets (10 mg/d for three days) one day before the procedure and two days after it + hydration with a infusion of 1000 mL of normal saline for 6 h prior to and after the procedure, G2: atorvastatin tablets (80 mg/d for three days), one day before the procedure and two days after it + hydration	G1: 95, G2: 94	hydration with a infusion of 1000 mL of normal saline for 6 h prior to and after the procedure	90	increase in serum creatinine of ≥ 0.3 mg/dL within 72 h after the procedure	elective coronary angiography	chronic kidney disease
Moghaddam et al., 2023	RCT	Iran	patients above 18 who underwent cardiac catheteriza- tion and had at least two risk factors of contrast nephropa- thy	allergic response to Nicorandil, low blood pressure dur- ing the hospitalization, severe kidney failure (GFR below 15 mL/min/173m2), cardiogenic shock, and history of con- sumption of metformin, dopamine, theophylline, sodium bicarbonate, diuretic, mannitol, and fenoldopam in the last 48 h before receiving the contrast agent.	344	oral Nicorandil (10 mg daily, three times) from 2 h before to 48 h after the catheterization + Intravenous normal saline (1 ml/kg/hr) was also given from 2 h before to 6 h after the pro- cedure.	172	received intravenous normal saline from 2 h before to 6 h after the procedure.	172	creatinine serum higher than 132.6 mol/L or higher than 25% of the baseline value 48 h after the procedure	cardiac catheterization	coronary problems (cardiovascular disease) and at least two risk factors of contrast nephropathy ((GFR) below 60 ml/min/1.73m2 or serum creatinine higher than 132.6 mol/L]) or diabetes
Yusuf et al., 2023	RCT	India	patients aged ≥ 18 years planned for elective PCI who had an estimated glomerular filtration rate (eGFR) between 30 and 89 mL/ min/1.73 m2	allergy to contrast agent, history of dialysis, NYHA IV heart failure, left ventricular ejection fraction (LVEF) < 30%, cardiogenic shock, malignancy and acute or chronic infection. Patients undergoing diagnostic CAG without therapeutic intervention, patients taking N-acetylcysteine or sodium bicarbonate and patients hav- ing received contrast agent within 7 days or either study drug within 1 month	315	G1: oral nicorandil (10 mg, 3 times/d) 1 day before procedure and for 2 days after PCI. G2: oral ranolazine (1000 mg, 2 times/d) 1 day before procedure and for 2 days after PCI.	G1:105, G2: 105	intravenous sodium chloride at a rate of 1.0 mL/kg/h (0.5 mL/kg/h for patients with LVEF < 45%) from 6 h before procedure till 12 h after procedure.	105	(rise in SCr ≥ 0.3 mg/dL within 48 h of procedure) after elective PCI	elective PCI	mild to moderate renal insufficiency, coronary artery disease
Fan et al., 2019	RCT	China	patients with renal dysfunction scheduled for coronary angiography or percutaneous coronary intervention (PCI)	end-stage renal disease; a history of kidney transplantation; left ventricular ejection fraction (LVEF) < 35% or New York Heart Association (NYHA) IV class; acute myocardial infarction; previous contrast media exposure within 1 week; allergy to contrast medium or nicorandil, and the administration of other medications, such as N-acetylcysteine, metformin, and sodium bicarbonate to prevent CIN.	265	10 mg of nicorandil three times per day (t.i.d.), from 2 days before to 2 days after an elective coronary procedure. +infusion of 0.9% saline at a rate of 1 mL/kg/h for 6 h before and 12 h after an elective coronary procedure.	133	intravenous infusion of 0.9% saline at a rate of 1 mL/kg/h for 6 h before and 12 h after an elective coronary procedure.	132	SCr increase of ≥ 25% and/or ≥ 0.5 mg/day within 72 h after exposure to the contrast medium	elective PCI	chronic Renal dysfunction was ((eGFR) ≤ 60 mL/min/1.73 m2)
Zeng et al., 2019	RCT	China	adult patients undergoing non-emergent PCI or coronary angiography (CAG)	pregnancy, lactation, used contrast agents within 1 week before PCI, allergy to contrast dye or use of low-permeability contrast agent, car- diovascular surgery or end-stage renal disease {creatinine clearance (Ccr) < 15 mL/min; Ccr=[140 − age] × weight (kg)/[0.818 × Scr (µmol/L)](× 0.85 if female)} or renal replacement, malignant neoplasms, balloon counterpulsation treatment (IABP); thyroid dysfunction, coagulopathy.	330	G1: double-dose group (30 mg/d) received nicorandil of about 10 mg diluted in 100 mL of 0.9% saline three times daily (beginning 2 days prior to the coronary intervention and continuing 2 days after it), 0.9% saline (at speed of 1.0 mL/kg/h, 0.5 mL/kg/h for patients with LVEF < 40%) intravenously administered from 12 h before to 12 h following the intervention. G2: usual dose (15 mg/d) Nicorandil of 5 mg and the same volume of hydration	G1: 111, G2: 107	0.9% saline (at speed of 1.0 mL/kg/h, 0.5 mL/kg/h for patients with LVEF < 40%) intravenously administered from 12 h before to 12 h following the intervention	112	as > 44.2 µmol/L (0.5 mg/dL) rise or > 25% elevation in serum creatinine concentration within 48 h after PCI compared with pre-PC.	non-emergent PCI or coronary angiography (CAG)	
Zhang et al., 2019 A	RCT	China	patients with moderate renal insufficiency aged > 18, patients with coronary artery disease who would subsequently undergoing an elective PCI and had 30 to 60 mL/min of Cr. clearance.	emergency PCI, allergy to contrast agent, severe renal insufficiency need for short- or long-term dialysis, or crCl < 30 mL/min, mL/min, New York Heart Association class IV, left ventricular ejection fraction (LVEF) < 30% or congestive heart failure, hypotension, recent exposure to contrast media within 2 weeks, electrolyte imbalance, cardiogenic shock, coagulopathy, thyroid dysfunction, neoplasm	250	(nicorandil orally 10 mg, 3 times/d) 1 day before operation and for 3 days after PCI + given intravenous sodium chloride at a rate of 1.0 mL/kg1/h1 (0.5/mL/kg/h1 for patients with LVEF < 45%) from 3 to 6 h before operation and 6 to 12 h after operation	125	1.0 mL/kg/h (0.5mL/kg/h for patients with LVEF < 45%) from 3 to 6 h before operation and 6 to 12 h after operation(at least 1000 mL hydration amount preoperatively and postoperatively).	125	increase in Scr levels by 0.5 mg/dL or 25% within 72 h after administration of the contrast media.	elective PCI	moderate renal insufficiency, coronary artery disease
Zhang et al., 2019 B	RCT	China	patients aged > 18, patients with coronary artery disease who would subsequently undergoing an elective PCI and had 30 to 60 mL/min of Cr. clearance.	emergency PCI, allergy to contrast agent, severe renal insufficiency need for short- or long-term dialysis, or crCl < 30 mL/min, mL/min, New York Heart Association class IV, left ventricular ejection fraction (LVEF) < 30% or congestive heart failure, hypotension, recent exposure to contrast media within 2 weeks, electrolyte imbalance, cardiogenic shock, coagulopathy, thyroid dysfunction, neoplasm	300	(nicorandil orally 10 mg, 3 times/d) 1 day before operation and for 3 days after PCI + of 1.0 ml/kg/h starting 12 h before, and ending 12 h after, PCI	150	sodium chloride was intravenously infused at a rate of 1.0 ml/kg/h starting 12 h before, and ending 12 h after, PCI	150	an increase in Scr levels by 0.5 mg/dL or 25% within 72 h after administration of the contrast media.	elective PCI	coronary artery disease
Iranirad et al., 2017	RCT	Iran	All adult (> 18 years) patients scheduled for PCI, existence of at least two of the following CIN risk factors, systolic heart failure (with documented ejection fraction < 40%), hypertension and diabetes mellitus (noted in their past medical history), age > 75 years and renal insufficiency	end-stage renal insufficiency (eGFR < 15 mL/min), acute renal insufficiency, pregnancy and lactation, pulmonary edema, cardiogenic shock, multiple myeloma, history of an allergic reaction to contrast agents or nicorandil, contrast media exposure within 7 days before the procedure, uremia, renal failure which led to having dialysis, and the administration of N-acetyl cysteine (NAC), metformin, dopamine, theophylline, sodium bicarbonate, mannitol, fenoldopam, diuretics, and nephrotoxic medicines within 48 h before the procedure.	128	10 mg nicorandil, daily from 30 min before to 3 days after the procedure and standard intravenous hydration (1 mL/kg/h) via normal saline, a maximum 100 mL/h for 2 h before and 6 h after the procedure,	64	intravenous hydration (1 mL/kg/h) via normal saline, a maximum 100 mL/h for 2 h before and 6 h after the procedure,	64	as an increase in SCr level at 44.2 µmol/L (0.5 mg/dL) or 25% above the baseline within 72 h after contrast medium administration without an alternative cause.	elective PCI	patients with at least 2 of : systolic heart failure (with documented ejection fraction < 40%), hypertension and diabetes mellitus, age > 75 years and renal insufficiency ( [eGFR] < 60 mL/min/1.73 m2, or baseline serum creatinine [SCr] > 1.5 mg/dL).
Fan et al., 2016	RCT	China	patients with renal insufficiency scheduled for coronary angiography	Cardiogenic shock, acute ST-segment elevated myocardial infarction requiring primary PCI, left ventricular ejection fraction (LVEF) < 30%, New York Heart Association (NYHA) IV class, allergy to contrast agent or nicorandil, previous contrast media exposure within 1 week, uremia, renal failure receiving dialysis and administration of N-acetylcysteine, metformin, or sodium bicarbonate within 48 h of the procedure.	240	10 mg nicorandil three times daily from 2 days before to 3 days after contrast media exposure,+ intravenous infusion of 0.9% saline at a rate of 1 mL/kg/h (0.5 mL/kg/h for patients with LVEF < 40%) at least 6 h before and 12 h after elective coronary procedure.	120	placebo + intravenous infusion of 0.9% saline at a rate of 1 mL/kg/h (0.5 mL/kg/h for patients with LVEF < 40%) at least 6 h before and 12 h after elective coronary procedure.	120	25% increase in SCr from baseline or 44 µmol/L (0.5 mg/dL) increase in absolute value within 72 h after exposure to contrast medium that is not attributable to any other identifiable cause	elective coronaray angiography/ PCI	Renal insufficiency (eGFR) ≤ 60 mL/min/1.73 m2 and coronary disease
Nawa et al., 2015	RCT	Japan	coronary artery disease (CAD) patients who would subsequently undergo elective PCI and who had a high cystatin C level, which was defined as a level greater than 0.95 mg/L in males and 0.87 mg/dL in females	end-stage renal failure on dialysis, a single functioning kidney, a history of kidney transplantation, hypotension with systolic blood pres- sures below 100 mmHg, acute myocardial infarction, acute heart failure, left ventricular ejection fraction (LVEF) less than 30% on echocardiogram or evidenced by pulmonary edema, multiple myeloma, pregnan- cy, a history of allergies to contrast medium or nicorandil, having received contrast medium within 7 days of study entry, having received an infusion of nicorandil within 1 month of study entry, parenteral use of diuretics, and the administration of N-acetylcysteine, metformin, sodium bicarbonate, teophiline, fenoldopam, mannitol, or a PDE-V inhibitor	213	2 vials of nicorandil (48 mg/V) dissolve in 100 mL 0.9% saline, and dripped it at speed of 0.1 mL/kg/h) plus 0.9% saline hydration intravenously infused at 1.0 mL/kg/h (initiated 4 h prior to elective PCI and were continued for 24 h after the procedure)	106	0.9% saline infusion 1.1 mL/kg/h	107	a 25% increase in serum creatinine or an increase in creatinine of 0.5 mg/dL from baseline at 48 h and at its maximum obtained within 1 month after the PCI.	elective PCI	poor renal function (non end stage CKD), coronary artery disease
Ko et al., 2013	RCT	Korea	Patients admitted with renal dysfunction [i.e., estimated glomerular filtration rate (eGFR) ≤ 60 mL/min by Cock-croft-Gault formula and serum creatinine (SCr) ≥ 1.1 mg/ dL] scheduled to undergo coronary angiography	< 18 years, acute myocardial infarction requiring primary or rescue coronary intervention, allergy to contrast dye or nicorandil, previous exposure to nicorandil or contrast me- dium within the preceding 7 days, pregnancy, left ventricu- lar ejection fraction (LVEF) < 30% by echocardiogram or evident by pulmonary edema, acute renal failure, renal fail- ure requiring dialysis, a single functioning kidney, history of kidney transplantation, life expectancy < 6 months, or the use of nonsteroidal anti-inflammatory drugs except for low dose aspirin, dopamine, mannitol, N-acetylcysteine,	173	12 mg nicorandil was diluted in 100 mL of 0.9% saline and administered intravenously over a 30-minute period just prior to coronary angiography. saline infusion is continued at least 8 h before and after procedure	85	100 mL of 0.9% saline was given by the same method. In all enrolled patients, an intravenous infu- sion of 0.45% saline at a rate of 1 mL/kg/hr (0.5 mL/kg/hr for patients with LVEF < 40%) was administered at least 8 h before and after an elective coronary procedure.	88	as > 0.5 mg/dL increase or > 25% rise in serum creatinine (SCr) concentration within 48 h of contrast exposure compared to baseline.	elective coronary angiography/ elective PCI	with renal dysfunction [i.e., (eGFR) ≤ 60 mL/min and serum creatinine (SCr) ≥ 1.1 mg/dL] with/ without coronary disease

**Table 2:** Baseline characteristics of the participants in the included studies. BMI: body mass index; eGFR: estimated glomerular filtration rate; scr: serum creatinine; N/A: not available

Study ID	Study arms	n	Age (years)	Sex (male)	BMI (Kg/m2)	Associated conditions		Baseline eGFR (mL/min)	SCr (mg/dL)	Contrast media volume (ml)	Cystatin C (mg/L)
			Mean ± SD	n (%)	Mean ± SD	HTN n (%)	DM n (%)	Mean ± SD	Mean ± SD	Mean ± SD	Mean ± SD
Kebar et al., 2023	Nicorandil	90	57.36 ± 8.74	57 (63.3)	N/A	52 (57.8)	46 (51.1)	44.79 ± 13.3	2.08 ± 1.18	N/A	N/A
	Control	90	58.52 ± 7.59	54 (60)	N/A	63 (70)	38 (42.2)	44.28 ± 13.43	2.09 ± 1.03	N/A	N/A
Moghaddam et al., 2023	Nicorandil	172	60.97 ± 9.68	78 (45.34)	27.19 ± 4,25	115 (66.86)	99 (57.55)	78.91 ± 28.65	0.95 ± 0.24	0.95 ± 0.24	N/A
	Control	172	60.61 ± 10.17	72 (41.86)	27.31 ± 4٫33	111 (64.53)	107 (62.21)	83.54 ± 28.81	0.91 ± 0.25	0.91 ± 0.25	N/A
Yusuf et al., 2023	Nicorandil	105	60.33 ± 8.731	92 (87.6)	N/A	30 (28.6)	37 (35.2)	63.64 ± 9.088	1.268 ± 0.16	1.268 ± 0.16	N/A
	Ranolazine	105	60.23 ± 8.830	94 (89.5)	N/A	40 (38.1)	42 (40.0)	63.16 ± 10.278	1.286 ± 0.182	1.286 ± 0.182	N/A
	Control group	105	60.46 ± 8.175	87 (82.9)	N/A	44 (41.9)	41 (39.0)	60.26 ± 10.781	1.323 ± 0.181	1.323 ± 0.181	N/A
Fan et al., 2019	Nicorandil	127	62.25 ± 16.63	76 (59.84)	24.35 ± 5.87	68 (53.54)	81 (63.78)	59.32 ± 19.31	1.37 ± 0.25	1.37 ± 0.25	1.17 ± 0.28
	Control	125	65.87 ± 17.62	67 (53.60)	23.78 ± 5.98	62 (49.6)	75 (60)	61.75 ± 22.56	1.34 ± 0.3	1.34 ± 0.3	1.12 ± 0.29
Zeng et al., 2019	Double-dose group	111	65.37 ± 7.19	78 (70.2)	24.67 ± 3.10	42 (37.8)	19 (17.1)	N/A	0.89 ± 0.205	0.89 ± 0.205	0.93 ± 0.22
	Usual-dose group	107	67.09 ± 6.85	73 (68.2)	24.85 ± 2.63	69 (64.5)	21 (19.6)	N/A	0.88 ± 0.112	0.88 ± 0.112	0.95 ± 0.25
	Control group	112	66.69 ± 7.33	67 (39.8)	24.60 ± 3.34	59 (52.7)	18 (16.1)	N/A	0.86 ± 0.163	0.86 ± 0.163	0.95 ± 0.20
Zhang et al., 2019 A	Nicorandil	125	67.4 ± 6.6	93 (74.4)	24.9 ± 2.2	N/A	24 (19.2)	51.2 ± 4.1	1.378 ± 0.18	1.378 ± 0.18	N/A
	Control	125	67 ± 7.2	89 (71.2)	25.1 ± 2	N/A	29 (23.2)	51 ± 3.8	1.374 ± 0.17	1.374 ± 0.17	N/A
Zhang et al., 2019 B	Nicorandil	150	67.25 ± 6.42	118 (78.7)	24.8 ± 2.17	69 (46)	34 (22.7)	N/A	1.401 ± 0.171	1.401 ± 0.171	6.85 ± 1.63
	Control	150	67.11 ± 7.19	114 (76)	25.1 ± 2.02	71 (47.3)	35 (23.3)	N/A	1.396 ± 0.162	1.396 ± 0.162	6.77 ± 1.78
Iranirad et al., 2017	Nicorandil	64	61.35 ± 11.77	39 (60.9)	28.43 ± 5.6	35 (54.7)	27 (42.2)	76.39 ± 24.6	1.0859 ± 0.22	1.0859 ± 0.22	N/A
	Control	64	57.64 ± 12.42	40 (62.5)	27.78 ± 4.8	41 (64.1)	26 (40.6)	83 ± 28.1	1.0359 ± 0.15	1.0359 ± 0.15	N/A
Fan et al., 2016	Nicorandil	120	66.07 ± 6.37	88 (73.33)	22.36 ± 2.19	69 (57.50)	66 (55.00)	49.62 ± 5.38	1.397 ± 0.1218	1.397 ± 0.1218	1.37 ± 0.38
	Control	120	67.37 ± 6.33	95 (79.17)	22.28 ± 2.98	74 (61.67)	62 (51.67)	50.38 ± 5.74	1.391 ± 0.1175	1.391 ± 0.1175	1.41 ± 0.42
Nawa et al., 2015	Nicorandil	106	70.4 ± 7.7	80 (81.6)	23.4 ± 3.4	69 (70.4)	57 (58.2)	59.6 ± 16.5	0.99 ± 0.29	0.99 ± 0.29	1.33 ± 0.33
	Saline	107	70.1 ± 8.1	74 (78.7)	23.5 ± 2.9	68 (72.3)	47 (50.0)	58.1 ± 16.4	1.02 ± 0.35	1.02 ± 0.35	1.35 ± 0.43
Ko et al., 2013	Nicorandil	73	70.8 ± 9.6	53 (72.6)	24.1 ± 3.2	57 (78.1)	30 (41.1)	37.5 ± 13.4	1.73 ± 0.6	1.73 ± 0.6	1.42 ± 0.51
	Control	76	69.1 ± 10.3	51 (67.1)	24.8 ± 3.7	61 (80.3)	42 (55.3)	40.1 ± 13.9	1.61 ± 0.44	1.61 ± 0.44	1.35 ± 0.47

**Table 3:** Safety outcomes analysis

Adverse event	No. of studies	No. of participants	RR [95% CI]	*P*-value	Heterogeneity
ESKD/Dialysis	5	1297	-0.19 [-0.37, -0.02]	*P* = 0.03	Chi² = 37.22, (P ,0.00001); I² = 89%
Cardiac death	5	1161	0.54 [0.17, 1.71]	*P* = 0.29	Chi² = 1.44, (*P* = 0.84); I² = 0%
Stroke	6	1401	1.04 [0.35, 3.10]	*P* = 0.95	Chi² = 3.04, (*P* = 0.69); I² = 0%
MI	5	1265	1.16 [0.44,3.10]	*P* = 0.76	Chi² = 2.18, (*P* = 0.70); I² = 0%
Conversion to emergency PCI	4	851	0.58 [0.20, 1.66]	*P* = 0.31	Chi² = 1.95, (*P* = 0.58); I² = 0%
Acute HF	4	1012	0.68 [0.30, 1.51]	*P* = 0.34	Chi² = 1.94, (*P* = 0.59); I² = 0%

### Risk of bias in studies and certainty of evidence

The risk of bias of included double-armed parallel clinical trials was assessed using the ROB 2 tool, considering five main domains. The evaluation of bias for the included clinical trials revealed that eight studies had some concerns for bias, while Fan 2019, Zhang 2019 (A), and Moghaddam 2023 exhibited a high risk in the fourth domain. Figure S1 shows detailed illustration of the risk of bias of double-armed parallel clinical trials.

Furthermore, certainty of evidence by the GRADE approach for study outcomes ranged from high to low quality of evidence. The incidence of CIN showed high certainty of findings, suggesting significant role for nicorandil in CIN reduction. Renal parameters and adverse events showed moderate to severe quality of evidence as shown in Table S2 in the supplementary file.

### Primary outcome

#### Contrast-induced nephropathy (CIN)

**Fig. 2:**
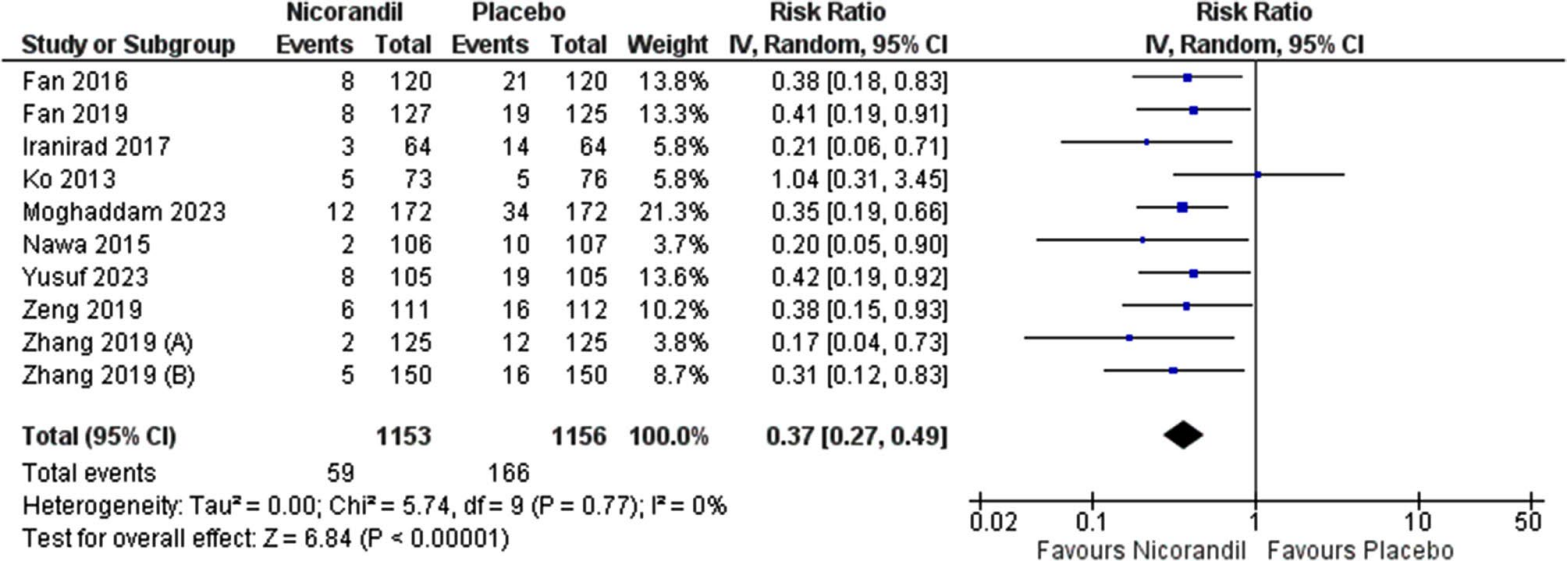
Forest plot showing pooled analysis for incidence of contrast-induced nephropathy (CIN) after interventions

**Fig. 3:**
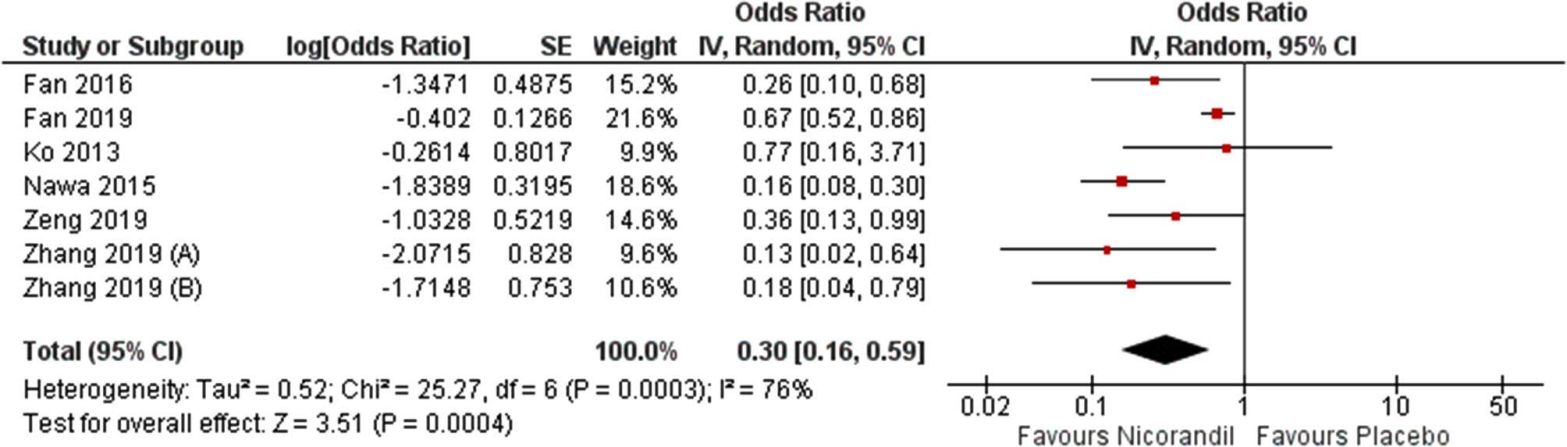
Forest plot showing incidence of contrast-induced nephropathy after adjusting multiple covariates by a logistic regression

**Fig. 4:**
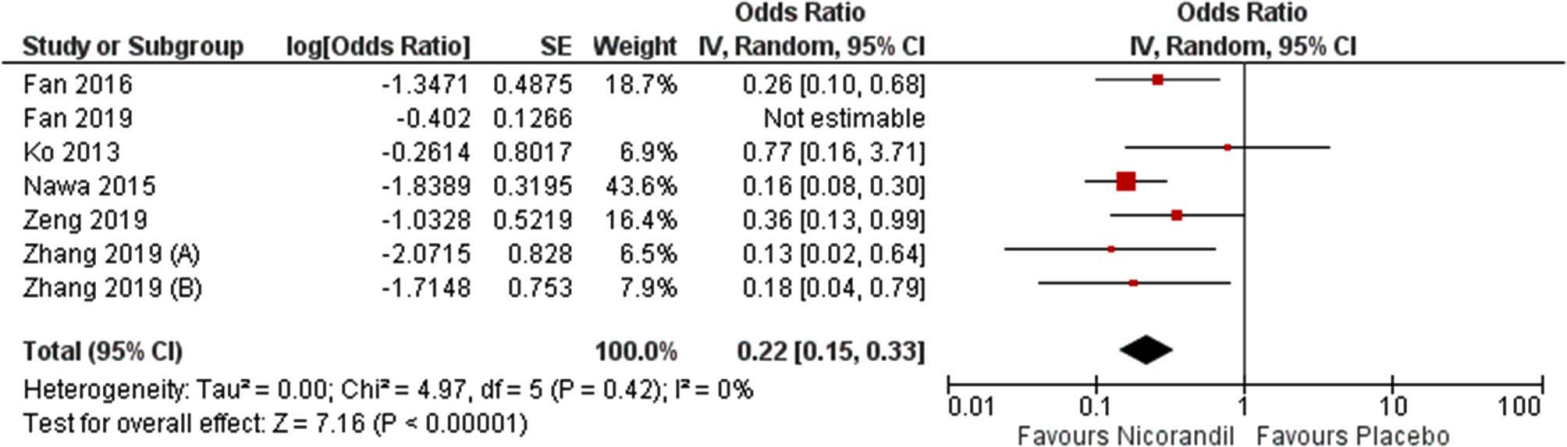
Forest plot showing pooled analysis for incidence of contrast-induced nephropathy (CIN) after exclusion for Fan et al., 2019

Meta-analysis results revealed that Nicorandil could significantly decrease CIN compared to control group (crude RR = 0.37, 95% CI [0.27 to 0.49], *P* < 0.001). No heterogeneity was detected (I² = 0.0%, *P* = 0.77) (Fig. 2). By adjusting for multiple covariates by a logistic regression, results remained significant (adjusted OR = 0.30, 95% CI [0.16 to 0.59], *P* = 0.0004) (Fig. 3). Pooled studies were heterogeneous (I² = 76%, *P* = 0.003). Heterogeneity was resolved after excluding Fan 2019 from analysis (adjusted OR = 0.22, 95% CI [0.15 to 0.33], P = < 0.001, I² = 0.0%, *P* = 0.42) (Fig. 4). We excluded Fan 2019. Due to the risk of bias with open-label conduct, different hydration methods, nicorandil doses and contrast volumes.

**Fig. 5:**
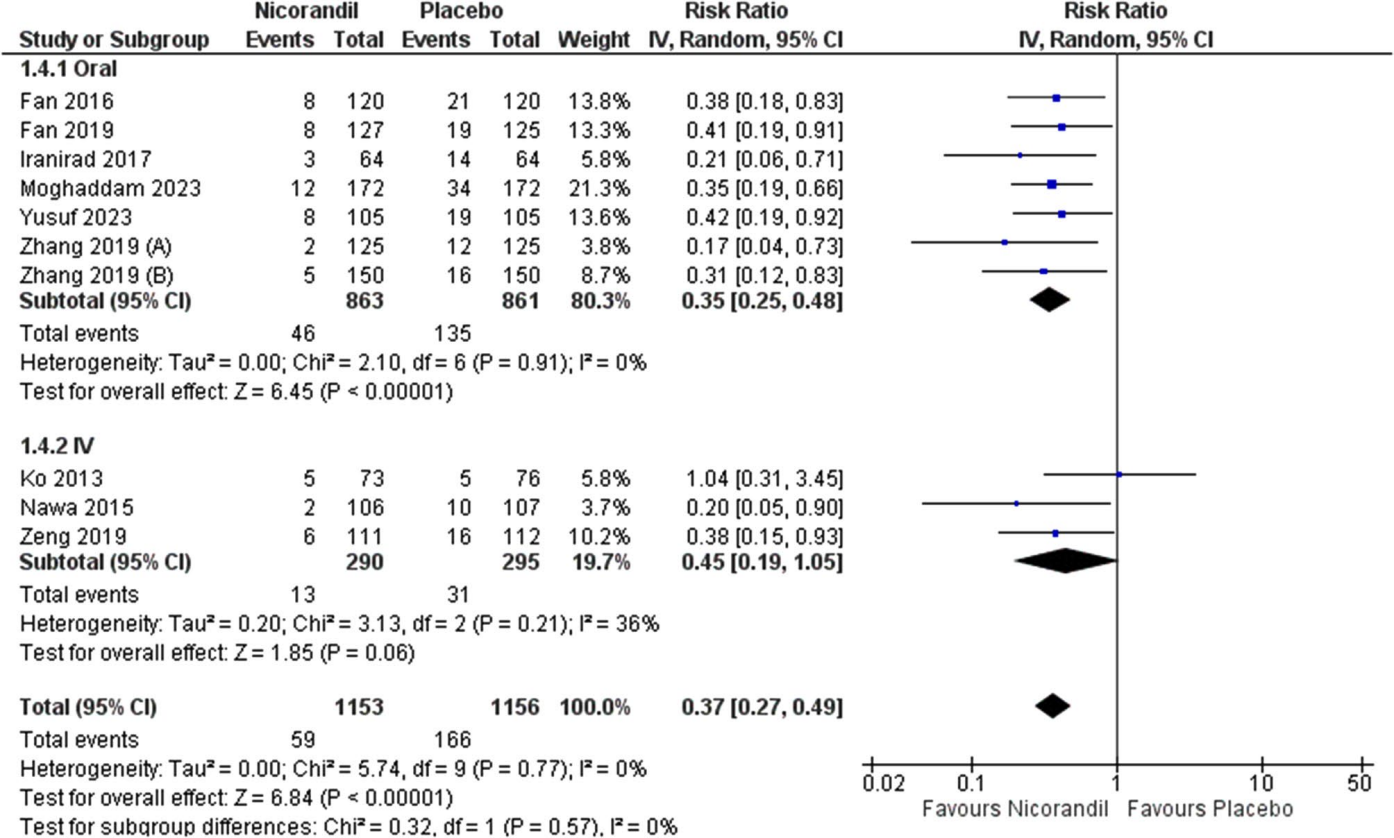
Forest plot showing subgroup analysis for incidence of contrast-induced nephropathy (CIN) according to method of administration into oral or intravenous (IV) injection

A subgroup analysis was performed according to whether the drug was taken oral or IV. There was a statistically significant difference between Nicorandil and control groups in the oral subgroup (RR = 0.35, 95% CI [0.25 to 0.48], *P* < 0.001, I² = 0.0%, *P* = 0.91). However, this effect appeared to be almost statistically significant in the IV subgroup (RR = 0.45, 95% CI [0.19 to 1.05], *P* = 0.06, I² = 36%, *P* = 0.21) (Fig. 5). However, there was no satistically significant difference between the two subgroups (*P* = 0.57).

**Fig. 6:**
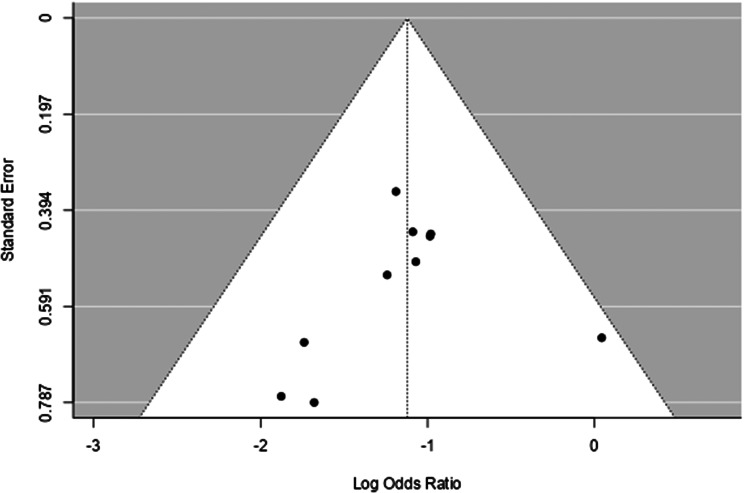
Funnel plot for risk of publication bias for primary outcome (CIN)

A funnel plot was utilized to visualize the presence of publication bias. The funnel plot was symmetrical indicating low risk of publication bias (Fig. 6). By assesing the presence of small study effect, egger regession test was used. There were no statistically significant small-study effects for the effect of nicorandil on CIN incidence upon analysis using egger regression test (*P* = 0.571).

### Secondary outcomes

#### Serum creatinine after 24, 48 and 72 h

Nicorandil group had significantly reduced the rise in serum creatinine level compared to control group at 24, 48 and 72 h (MD= -4.45 umol/L, 95% CI [-7.94 to -0.97], *P* = 0.01), (MD= -5.57 umol/L, 95% CI [-8.95 to -2.20], *P* < 0.001) and (MD= -5.70 umol/L, 95% CI [-9.57 to -1.82], *P* = 0.004) respectively. Heterogeneity was detected across the three endpoints (I² = 69%, *P* = 0.02), (I² = 81%, *P* < 0.001), and (I² = 90%, *P* < 0.001), respectively (Figures S2–4). A subgroup analysis was performed according to the effect size on each day, which revealed that there was no statistically significant difference between the effects across the three days (*P* = 0.87), ranging from (ES= -5.70, -4.45) (Figure S5).

#### eGFR (ml/min/ 1.73 m2) after 24, 48, and 72 h

There was no statistically significant difference between Nicorandil group and control group regarding eGFR measured at 24, 48 and 72 h (MD = 2.17, 95% CI [-2.03 to 6.37], *P* = 0.31), (MD = 1.89, 95% CI [-0.15 to 3.93], *P* = 0.17) and (MD = 2.26, 95% CI [-0.37 to 4.89], *P* = 0.09) respectively. Heterogeneity was detected across the three outcomes (I² = 89%, *P* = 0.001), (I² = 91%, *P* < 0.001) and (I² = 93%, *P* < 0.001) respectively (Figures S6–8). A subgroup analysis was performed according to the effect size on each day, which revealed that there was no statistically significant difference between the effects across the three days (*P* = 0.97); ranging from (ES = 1.89, 2.26) (Figure S9).

#### Serum cystatin C (mg/L) after 24, 48, and 72 h

There was no statistically significant difference between the Nicorandil group and the control group regarding serum Cystatin C level measured at 24 h after intervention (MD= -0.08, 95% CI [-0.21 to 0.06], *P* = 0.25). Pooled studies were heterogeneous (I² = 81%, *P* = 0.002) (Figure S10). However, Cystatin C level was significantly lower in the Nicorandil group compared to control group at 48 and 72 h (MD= -0.34, 95% CI [-0.54 to -0.13], *P* = 0.001) and (MD= -0.19, 95% CI [-0.37 to 0], *P* = 0.05) respectively. Heterogeneity was detected across the two outcomes (I² = 93%, *P* < 0.001) and (I² = 93%, *P* < 0.001) respectively (Figures S11,12). A subgroup analysis was performed according to the effect size in each day that revealed that there was no statistically significant difference between the effects across the three days (*P* = 0.10); ranging from (ES= -0.34, -0.08) (Figure S13).

#### Serum BUN level (mmol/L)

Increase in BUN level was statistically significant lower in Nicorandil group compared to the control group (MD= -0.19, 95% CI [-0.37 to -0.02], *P* < 0.001). Mild heterogeneity was detected across studies (I² = 41%, *P* = 0.16) (Figure S14).

### Adverse events

There was no statistically significant difference between the Nicorandil group and the control group regarding adverse events, including end-stage kidney disease (ESKD)/dialysis, cardiac death, stroke, myocardial infarction, conversion to emergency PCI, and acute heart failure. All outcomes were homogeneous. A summary of the Adverse events observed during studies is found in the Table 3 and the figures of the forest plots in the supplementary file (Figure S15–20).

### Leave one out (sensitivity) and subgroup analysis

Sensitivity analysis was conducted on all outcomes by omitting one study each time to evaluate the robustness of the results. CIN, BUN, and adverse events were robust, and the results did not change by removing any of the included studies (Figures S 21–28). Regarding serum creatinine, eGFR, and Cystatin C, the significance changed by removing certain studies (Figures S29–37). A subgroup analysis was conducted for our primary outcome based on the method of administration and for the secondary outcomes based on different endpoints as mentioned previously.

### Meta-regression analysis

Multiple linear regression including multiple predictor variables, was conducted for CIN, serum creatinine, and eGFR. None of the covariates showed a significant association with CIN (Table S3). The estimated effects were: baseline creatinine β = 0.7413 (SE = 0.6758, *p* = 0.272), baseline eGFR β=−0.0147 (SE = 0.0131, *p* = 0.264), contrast volume β=−0.0095 (SE = 0.0072, *p* = 0.1839), age β = 0.0256 (SE = 0.0467, *p* = 0.58), and BMI β=−0.0624 (SE = 0.0959, *p* = 0.515). No predictors for serum creatinine reached statistical significance (Table S4). The estimates were: age β=−0.3634 (SE = 0.318, *p* = 0.2532), BMI β = 0.1828 (SE = 1.11, *p* = 0.87), contrast volume β=−0.0526 (SE = 0.077, *p* = 0.495), baseline eGFR β=−0.0564 (SE = 0.1121, *p* = 0.6147), and baseline creatinine β = 3.6281 (SE = 3.6867, *p* = 0.3251). In Table S5 (predicting eGFR), again, there were no statistically significant associations. The effects were: age β = 0.1194 (SE = 0.4, *p* = 0.7653), BMI β = 0.8685 (SE = 1.12, *p* = 0.4368), baseline creatinine β=−3.7378 (SE = 3.46, *p* = 0.28), baseline eGFR β = 0.1112 (SE = 0.1118, *p* = 0.3198), and contrast volume β = 0.051 (SE = 0.139, *p* = 0.72).

Overall, across all three models, age, BMI, contrast volume, baseline Serum Creatinine, and baseline eGFR were not significant predictors of CIN, serum creatinine, or eGFR, indicating no clear study-level modifiers in these analyses.

## Discussion

The use of iodinated contrast agents during cardiac procedures is associated with an acute deterioration of renal function, which exacerbates morbidity and mortality in patients with CKD [23]. In this systematic review and meta-analysis, we focused on elucidating the effectiveness of Nicorandil in preventing CIN and enhancing renal function parameters, including serum creatinine, eGFR, serum cystatin C, and BUN level, in patients receiving contrast agents for PCI or cardiac catheterization. Based on the results of 11 RCTs with a participation of 2,837 patients, there was a significantly lower incidence of CIN among patients with CKD who had to undergo PCI or angiography and received Nicorandil as additional therapy alongside with traditional hydration therapy compared to those who received hydration alone in control group, underscoring the role of Nicorandil on renal function. These findings are consistent with current literature since Butt et al., reported reduced risk of CIN among patients undergoing cardiac procedures who received additional Nicorandil compared to the control group [46]. Similarly, Pranata et al. and Waseem et al., noticed significant reduction in the incidence of CIN among nicorandil groups [23, 47]. These insights highlight the crucial role of Nicorandil for patients at high risk of renal dysfunction undergoing coronary procedures.

On performing subgroup analysis according to the method of Nicorandil administration, both groups showed clinical significance, evidenced by reduced incidence of CIN in the intervention groups. However, intravenous Nicorandil did not reach statistical significance. These variations are attributed to the absence of a standardized protocol for intravenous administration and the variability of hydration volumes across studies [48]. Additionally, Ko et al., 2013 [31] study showed that intravenous Nicorandil did not significantly reduce the risk of CIN compared to the hydration group, while Zeng et al., 2019 [43] who showed that Nicorandil 15 mg/dl showed a non-statistically significant difference, unlike the dose of 30 mg/dl, which revealed a significant difference compared to hydration alone. Moreover, there are no well-designed studies that directly compare between oral and intravenous administration of Nicorandil in patients undergoing cardiac interventions. These findings underscore the need for a standard protocol for oral and intravenous administration of Nicorandil before procedures.

Notably, the pooled analysis of renal parameters showed that Nicorandil had significantly reduced serum creatinine levels at 24, 48, and 72 h after the cardiac procedure compared to the control group. On the other hand, the outcome of eGFR was not significantly different between groups across the three days after coronary intervention. These findings are consistent with Khan et al., who reported similar results with a significant reduction in serum creatinine, while eGFR values were non-significant between study groups [49]. The most plausible explanation for variations in renal parameters is limited power at each time-point plus substantial between-study heterogeneity. At 24 h, only 3 studies were included, with an imprecise pooled effect (*p* = 0.31 and wide CI). At 48 h after intervention, evidence based on 6 studies and the pooled effect was almost significant (*p* = 0.07) with the narrowest CI, consistent with a power effect rather than a true absence of treatment effect. Across all time-points, heterogeneity was high (I² = 89–93%), which further dilutes statistical significance. Moreover, Variations in measurement methods also may affect the non-significant results: the included trials used different kidney-function metrics and equations, and eGFR equations are non-linear and assume steady state. Small changes in creatinine can translate inconsistently into eGFR changes depending on the formula, baseline creatinine, and calibration, especially within 72 h, when steady state may not hold [50].

There was no statistical difference in serum Cystatin C levels between the Nicorandil and control groups at 24 h; the mean difference was − 0.08. However, the Nicorandil group had significantly lower Cystatin C levels at 48 and 72 h compared with the control group. Subgroup analysis according to daily effects demonstrated a non-significant result across the three days. The level of BUN was statistically significantly lower in the Nicorandil group compared to the control group; however, this difference was modest. Moreover, across three meta-regression models, none of the study-level covariates, including age, BMI, baseline Serum creatinine, baseline eGFR, or contrast volume, significantly explained between-study differences in CIN incidence or in post-procedure creatinine/eGFR changes (all *p* > 0.05), and the effect directions were small and inconsistent. Clinically, this suggests that within the ranges and contemporary practices represented in the included studies, such as modern low-osmolar agents, routine hydration, and avoidance of extreme contrast dosing, the risk of CIN and short-term renal biomarker shifts is not meaningfully modified by these common baseline characteristics at the study level.

Interestingly, Nicorandil showed a good safety profile, as no significant differences in adverse events, including myocardial infarction, stroke, and heart failure, were noted between the Nicorandil and control group. This buttresses the possibility of the use of Nicorandil in actual patients undergoing PCI and CAG or even in those patients with cardiovascular risk factors. Utilizing meta-regression analysis on studies and their key characteristics that influence the treatment outcomes, we identified age, baseline serum creatinine, and eGFR as factors that independently predicted renal outcomes in patients receiving Nicorandil. A recent systematic review [51] specifically concentrated on the cardiac impact of Nicorandil in patients with PCI. While this systematic review helped understand the cardioprotective therapy of Nicorandil, it did not provide much information about the renal impact of Nicorandil. It showed how Nicorandil contributed to the reduction of myocardial infarction and other cardiac events. However, this study created a gap in the overall profile of nephroprotective effects of this drug to be filled.

### Previous literature

A meta-analysis by Pranata et al. in 2020 [23] for CKD patients undergoing PCI showed similar results about the efficacy of Nicorandil in the reduction of CIN incidence, but it lacked comprehensive insights about other kidney parameters like serum creatinine, BUN, and eGFR with a limited number of RCTs. In Li et al. 2018, it was discovered that Nicorandil was linked to a substantial decrease in the occurrence of CIN, as well as reductions in serum creatinine and cystatin C levels. However, a primary limitation of this study was the small sample size, as it only included four RCTs. Mei et al. 2022 indirectly compared Nicorandil on the prevention of CIN with peptide therapies and provided a relatively smaller analysis than the present review, Still, unfortunately, they did not review the other renal parameters such as eGFR and serum cystatin C, similar to Butt et al. [46, 52]. In addition, they did not evaluate the adverse events of Nicorandil in patients enrolled in the PCI or CAG in terms of cardiovascular outcomes.

### Clinical implications

We found that managing CIN carries significant clinical implications for patient care, particularly for those at high risk. Because of the substantial reduction achieved with Nicorandil, this agent could be considered as an adjuvant measure to hydration in the patient scheduled for PCI or CAG, particularly in CKD or other predisposing circumstances to CIN. The combination of data and abilities to exert a direct vasodilating action and to induce ischemic preconditioning raises the hope of using Nicorandil to reduce both renal and cardiac complications in these patients. The decline in serum creatinine and cystatin C levels also supports the significant gains with Nicorandil use. In addition, based on the safety profile of Nicorandil and its aggregated cardiac protective nature, it could be prescribed to patients suffering from other cardiovascular diseases without a heightened risk of cardiac consequences. However, we should interpret these insights with caution due to heterogeneity of serum creatinine, cystatin C, and eGFR.

### Limitations and recommendations

The limitations of this systematic review include variance in dosing with potential differences in the presented results, and it would be challenging to set an ideal dose against CIN. However, there was heterogeneity in the timing of Nicorandil use; some studies started using it 2–3 days before the intervention and maintained it thereafter, while in others it was used only on the day of the intervention. Moreover, there was the variability of definitions for CIN across studies due to variability of guidelines over time. Future studies concerning the use of Nicorandil should seek to set a uniform dosing schedule and time within which Nicorandil can be administered to enhance the prevention of CIN. Therefore, subsequent research should seek to establish comparisons between Nicorandil and other pharmacological agents commonly utilized in CIN prevention, including N-acetylcysteine or statins, with direct head-to-head comparisons for uniform dosing regimens and long-term follow-up, to determine the best and effective preventive approaches to CIN among high-risk groups.

## Conclusion

In conclusion, Nicorandil significantly reduced the risk of CIN and was associated with protective effects on kidney function to be a potential safe option for the reduction of the incidence of CIN in high-risk patients undergoing cardiac interventions with caution on interpretation of eGFR and creatinine changes due to notable heterogeneity. Further large-scale studies are still required to determine the best dosing regimen for Nicorandil and its administration schedule to evaluate adverse renal effects in the long term and to compare the efficacy of Nicorandil with other pharmacologic agents.

## Supplementary Information

Below is the link to the electronic supplementary material.

Supplementary Material 1

## Data Availability

All data generated or analyzed during this study are included in this published article.
