# Supplementary material for: The protective effects of Nicorandil on renal function in patients undergoing coronary interventions: a systematic review and meta-analysis
Source: BMC Nephrol. 2025 Dec 1;26:680. doi: 10.1186/s12882-025-04564-8 (PMC12670811; doi:10.1186/s12882-025-04564-8)
Supplement: Supplementary file 1 — Supplementary Material 1 [file 12882_2025_4564_MOESM1_ESM.docx]

**Supplementary file**

1. **Search strategy**
2. **Supplementary Figures**
3. **GRADE assessment**
4. **Supplementary Tables**

**Search strategy for databases:** (searched September 15, 2024)

**PubMed**

("Nicorandil"[Mesh] OR nicorandil) AND (("Contrast-Induced Nephropathy"[Mesh]) OR (contrast nephropathy) OR ("contrast-related nephropathy") OR nephropathy OR ("kidney disease*"))

**Scopus:**

(TITLE-ABS-KEY (nicorandil) AND TITLE-ABS-KEY ("contrast induced nephropathy" OR "contrast-related nephropathy" OR nephropathy OR "kidney disease*"))

**Web of Science:**

TS=(nicorandil) AND TS=("contrast induced nephropathy" OR "contrast-related nephropathy" OR nephropathy OR "kidney disease*")

**Cochrane CENTRAL:**

nicorandil AND ("contrast induced nephropathy" OR "contrast-related nephropathy" OR nephropathy OR "kidney disease*")

**Supplementary Figures:**


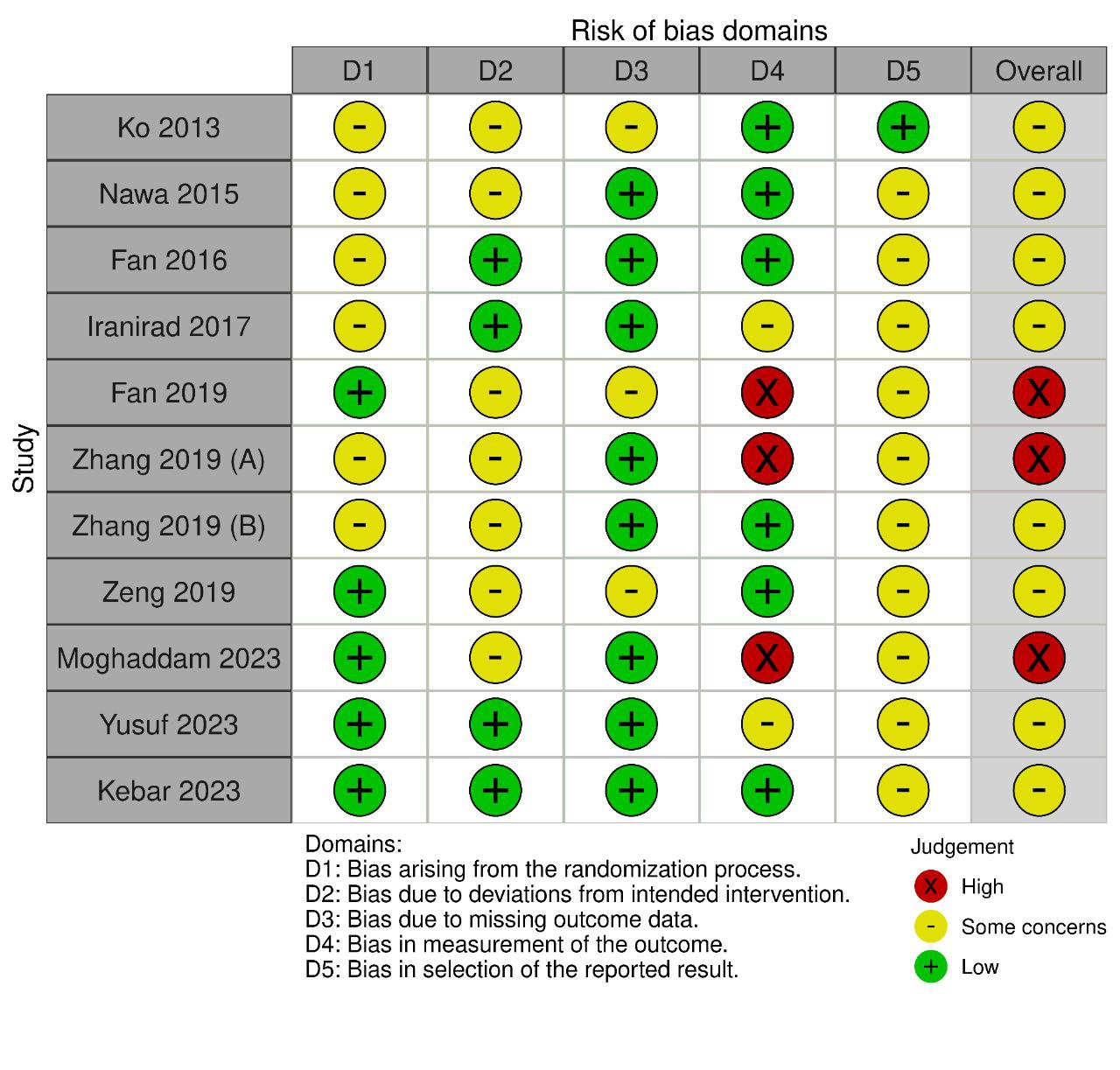


**Figure S1:** Summary of Risk of Bias assessment using (RoB2 tool)


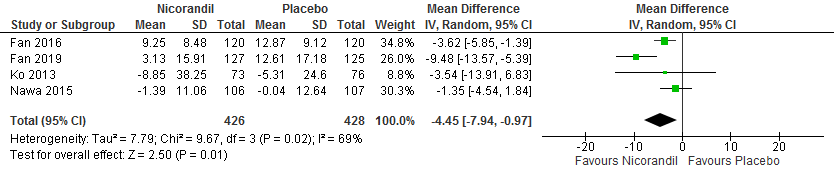


**Figure S2:** Forest plot showing changes in Serum creatinine after coronary interventions in both groups after 24 hours after interventions


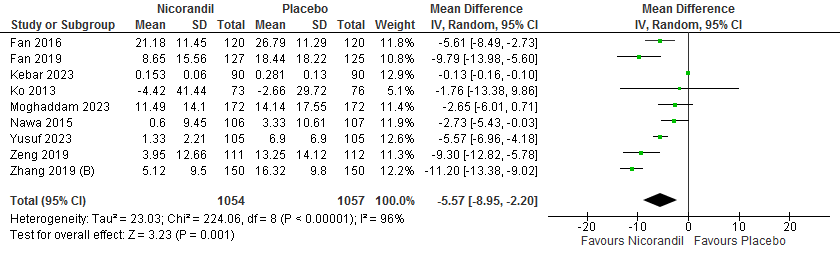


**Figure S3:** Forest plot showing changes in Serum creatinine after coronary interventions in both groups after 48 hours of interventions


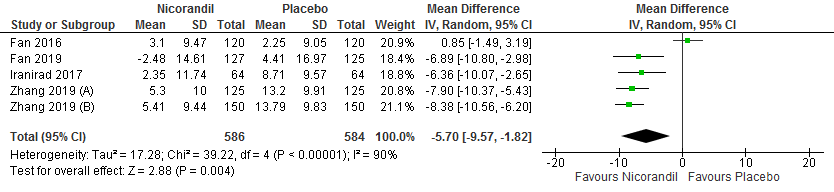


**Figure S4:** Forest plot showing changes in Serum creatinine after coronary interventions in both groups after 72 hours of interventions


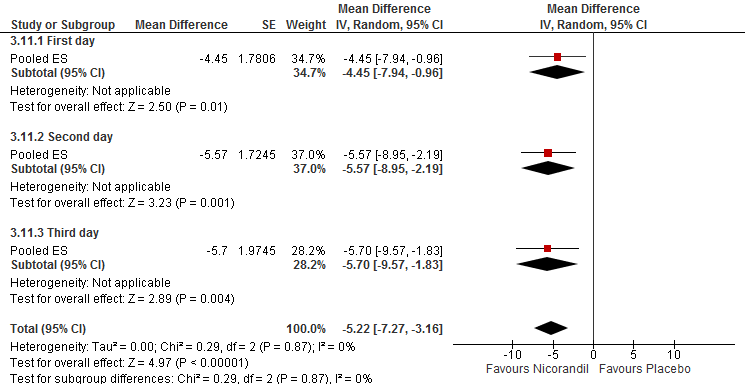


**Figure S5:** Forest plot showing Serum creatinine after coronary interventions with subgroup analysis according time of intervention


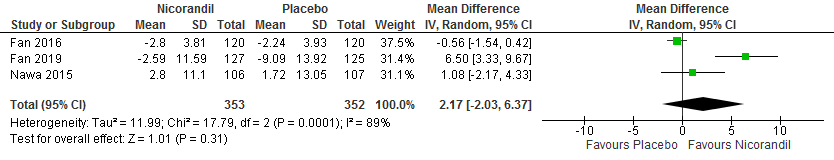


**Figure S6:** Forest plot showing changes in estimated glomerular filtration rate (eGFR) (ml/min/ 1.73 m2) after coronary interventions in both groups after 24 hours of interventions


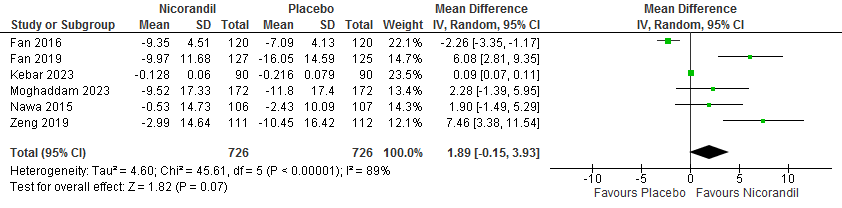


**Figure S7:** Forest plot showing changes in estimated glomerular filtration rate (eGFR) (ml/min/ 1.73 m2) after coronary interventions in both groups after 48 hours of interventions


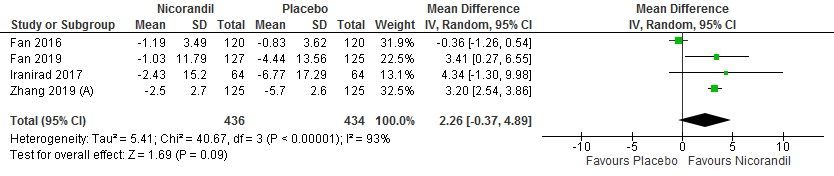


**Figure S8:** Forest plot showing changes in estimated glomerular filtration rate (eGFR) (ml/min/ 1.73 m2) after coronary interventions in both groups after three days


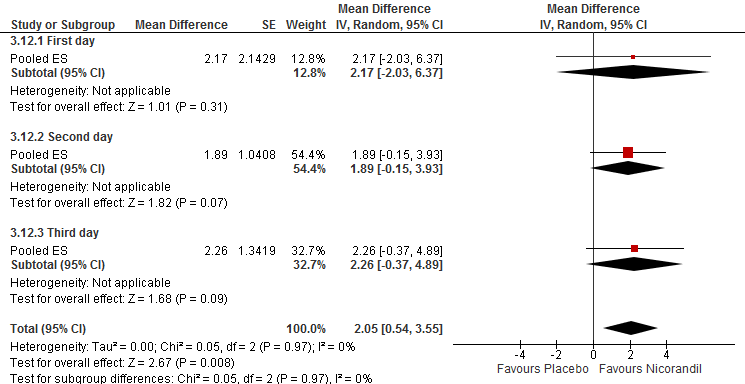


**Figure S9:** Forest plot showing eGFR changes after coronary interventions with subgroup analysis according time of intervention


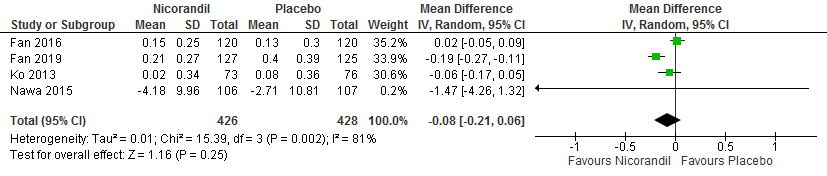


**Figure S10:** Forest plot showing changes in serum cystatin-C (mg/L) post-coronary interventions in both groups after 24 hours


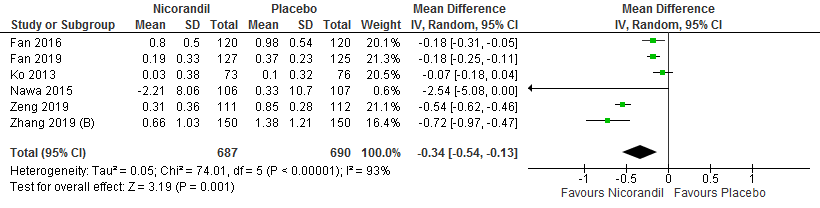


**Figure S11:** Forest plot showing changes in serum cystatin-C (mg/L) post-coronary interventions in both groups after 48 hours


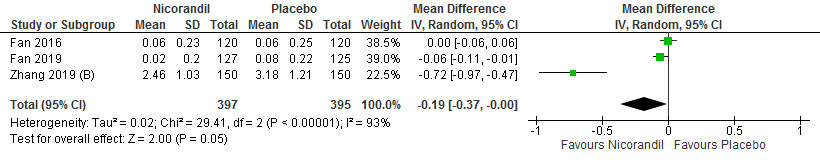


**Figure S12:** Forest plot showing changes in serum cystatin-C (mg/L) post-coronary interventions in both groups after 72 hours


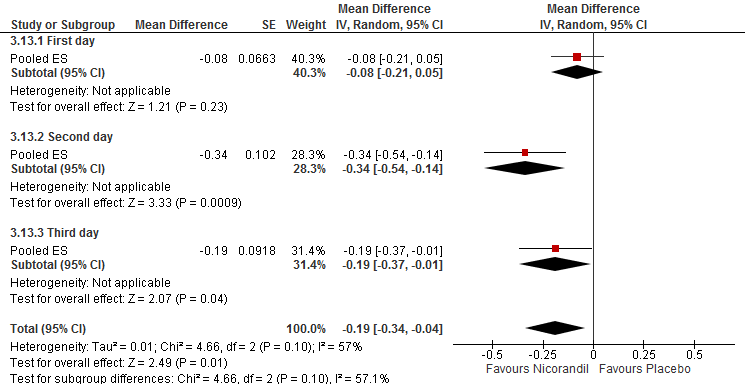


**Figure S13:** Forest plot showing changes in serum cystatin-C (mg/L) after coronary interventions with subgroup analysis according time of intervention


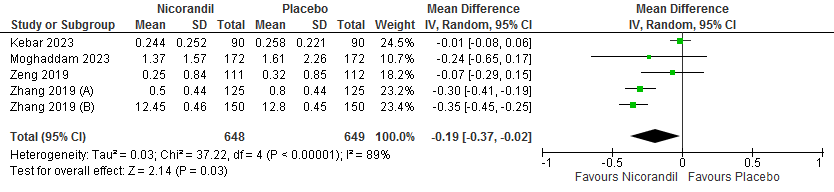


**Figure S14:** Forest plot showing Serum BUN level (mmol/L) in both groups


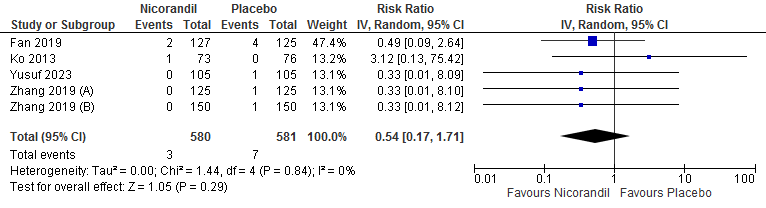


**Figure S15:** Forest plot showing proportion of patients underwent urgent dialysis due to worsening condition


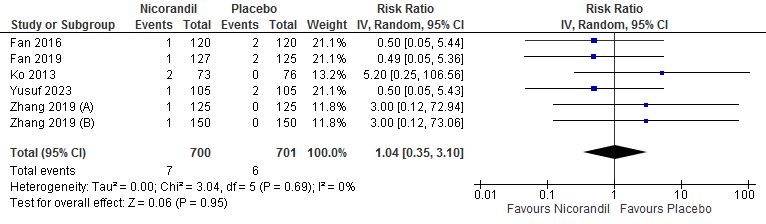


**Figure S16:** Forest plot showing incidence of cardiac death in both groups


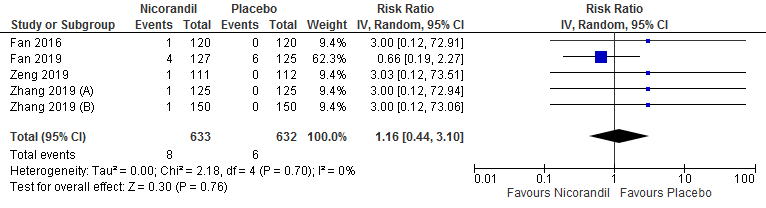


**Figure S17:** Forest plot showing incidence of cerebral stroke in both groups


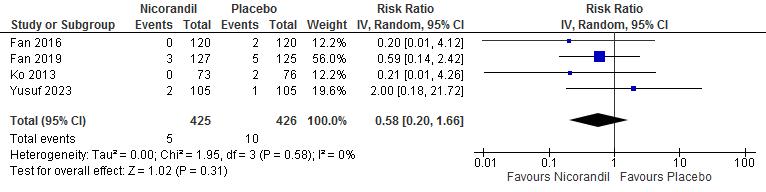


**Figure S18:** Forest plot showing incidence of myocardial infarction (MI) in both groups


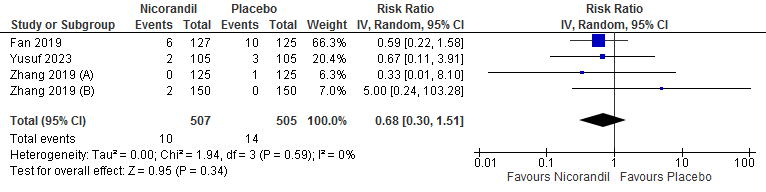


**Figure S19:** Forest plot showing conversion to emergency PCI


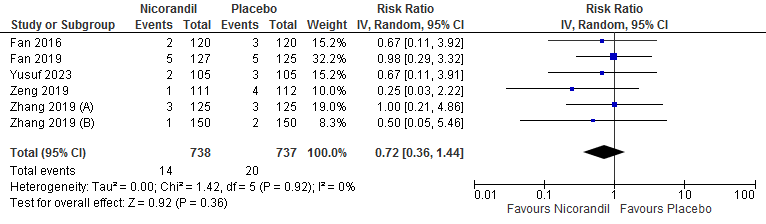


**Figure S20:** Forest plot showing incidence of heart failure in both groups

**Sensitivity analysis Figures**


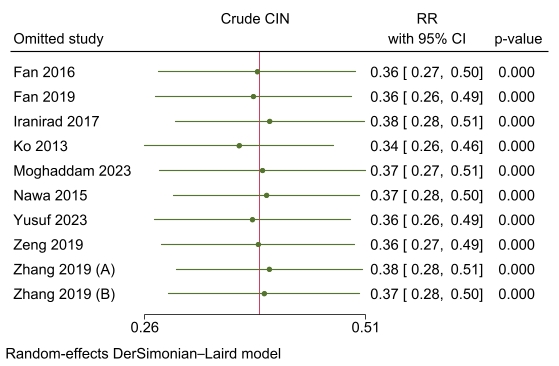


**Figure S21:** Sensitivity analysis of primary outcome (contrast induced nephropathy)


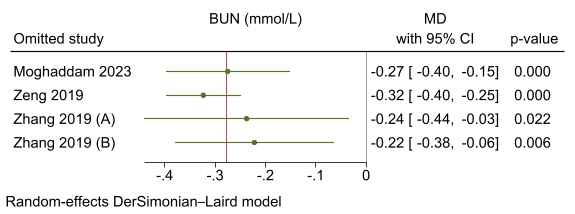


**Figure S22:** Sensitivity analysis of changes in serum BUN


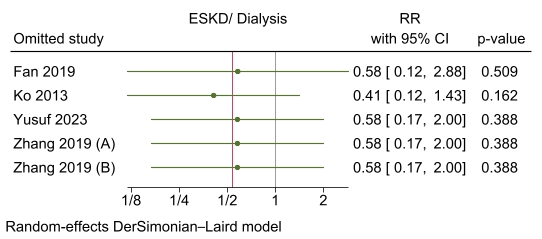


**Figure S23:** Sensitivity analysis the need for urgent dialysis


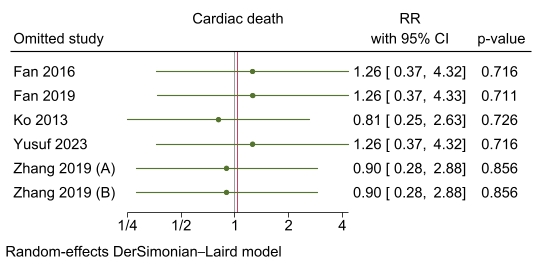


**Figure S24:** Sensitivity analysis for incidence of cardiac death


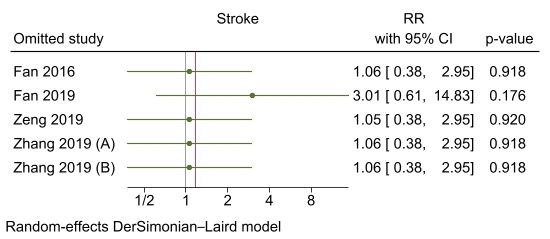


**Figure S25:** Sensitivity analysis of incidence of stroke


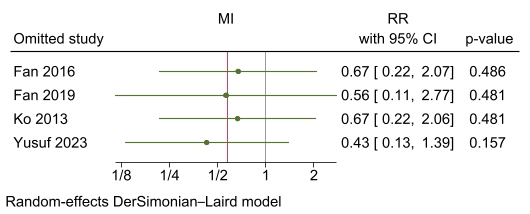


**Figure S26:** Sensitivity analysis of incidence of myocardial infarction (MI)


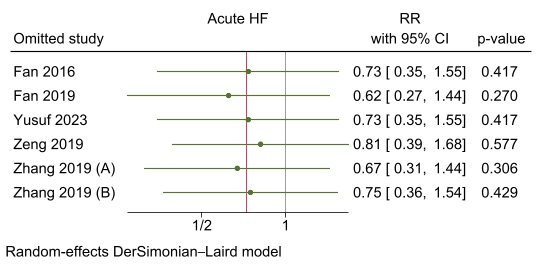


**Figure S27:** Sensitivity analysis for incidence of acute heart failure


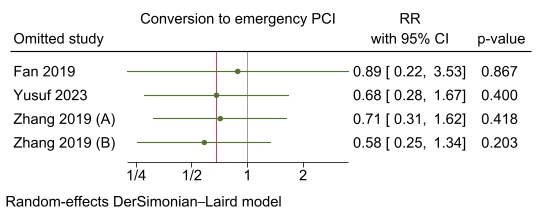


**Figure S28:** Sensitivity analysis for the proportion of patients underwent conversion PCI


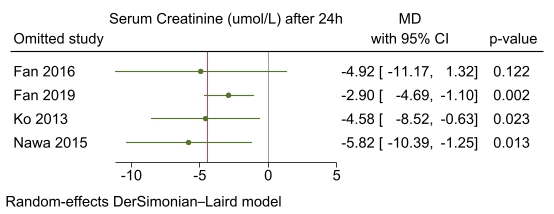


**Figure S29:** Sensitivity analysis of changes in serum creatinine after 24 hours


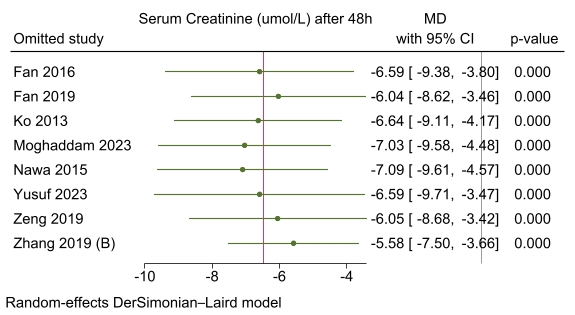


**Figure S30:** Sensitivity analysis of changes in serum creatinine after 48 hours


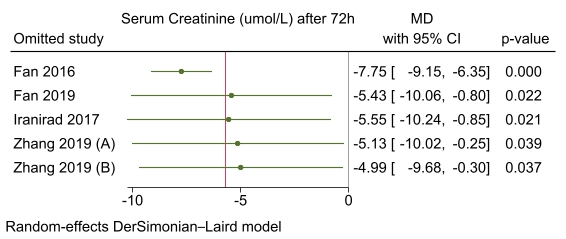


**Figure S31:** Sensitivity analysis of changes in serum creatinine after 72 hours


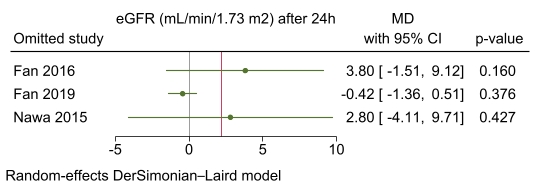


**Figure S32:** Sensitivity analysis of changes in eGFR after 24 hours


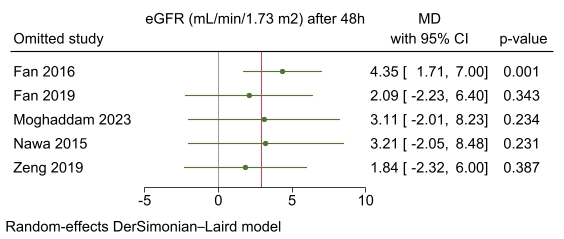


**Figure S33:** Sensitivity analysis of changes in eGFR after 48 hours


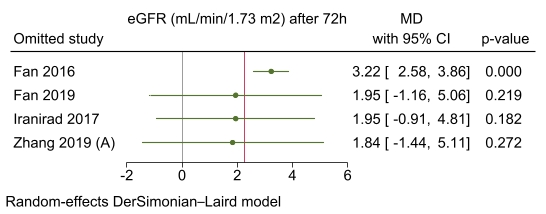


**Figure S34:** Sensitivity analysis of changes in eGFR after 72 hours


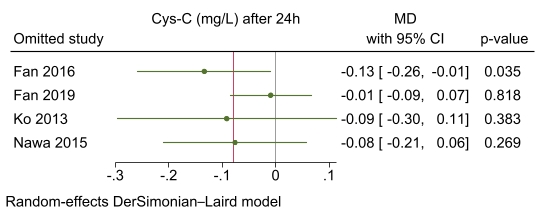


**Figure S35:** Sensitivity analysis of serum Cys-C after 24 hours


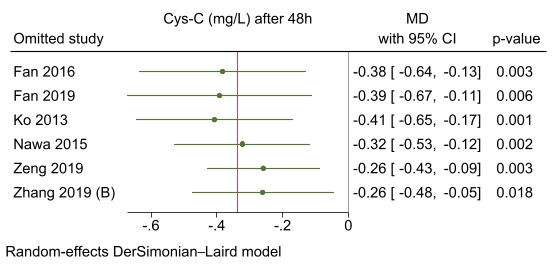


**Figure S36:** Sensitivity analysis of serum Cys-C after 48 hours


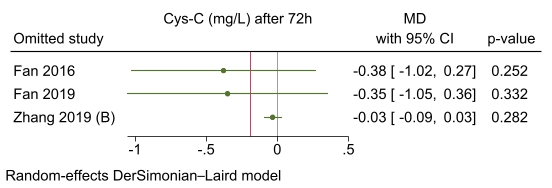


**Figure S37:** Sensitivity analysis of serum Cys-C after 72 hours

| **Outcome** | **No. of included studies** | **Study design** | **No. of participants in intervention group** | **No. of participants in control group** | **Effect size, 95% CI** | **heterogeneity** | **Risk of bias** | **inconsistency** | **imprecision** | **indirectness** | **Other considerations** | **Certainty of evidence** |
| --- | --- | --- | --- | --- | --- | --- | --- | --- | --- | --- | --- | --- |
| Contrast induced nephropathy (CIN) | 10 | RCT | 1153 | 1156 | aOR = 0.22, 95% CI [0.15, 0.33] | P = 0.42  I²= 0% | Serious* | Not serious | Not serious | Not serious | Symmetrical distribution with no publication bias (egger regression test, P= 0.571). | ⊕ ⊕⊕ ⊕  high |
| Change in serum creatinine after 24 h | 4 | RCT | 426 | 428 | -4.45, [-7.94, -0.97] | P = 0.02  I²= 69% | Serious* | Serious** | Not serious | Not serious | Not exist # | ⊕⊕◯◯  Low |
| Change in serum creatinine after 48 h | 9 | RCT | 1054 | 1057 | -5.57, [-8.95, -2.2] | P < 0.00001  I²= 96% | Serious* | Serious** | Not serious | Not serious | Not exist # | ⊕⊕◯◯  Low |
| Change in serum creatinine after 72 h | 4 | RCT | 586 | 584 | -5.70, [-9.57, -1.82] | P < 0.00001  I²= 90% | Serious* | Serious** | Not serious | Not serious | Not exist # | ⊕⊕◯◯  Low |
| Change in eGFR after 48 h | 3 | RCT | 726 | 726 | 1.89, [-0.15, -3.93] | P = 0.0001  I²= 89% | Serious* | Serious** | Not serious | Not serious | Not exist # | ⊕⊕◯◯  Low |
| Change Serum Cystatin C level | 6 | RCT | 1510 | 1513 | -0.19, [-0.34, -0.04] | P = 0.1  I²= 57% | Serious* | Serious** | Not serious | Not serious | Not exist # | ⊕⊕◯◯  Low |
| Cardiac death | 6 | RCT | 700 | 7001 | RR: 1.04, [0.35,3.1] | P = 0.69  I²= 0% | Serious* | Not serious | Not serious | Not serious | Not exist # | ⊕⊕⊕◯  Moderate |
| Cerebral stroke | 5 | RCT | 633 | 632 | R: 1.16, [0.44, 3.1] | P = 0.7  I²= 0% | Serious* | Not serious | Not serious | Not serious | Not exist # | ⊕⊕⊕◯  Moderate |
| Myocardial infarction | 4 | RCT | 425 | 426 | 0.58, [0.2, 1.66] | P = 0.58  I2 = 0% | Serious* | Not serious | Not serious | Not serious | Not exist # | ⊕⊕⊕◯  Moderate |
| Acute heart failure | 6 | RCT | 738 | 737 | 0.72, [0.36, 1.44] | P = 0.92  I2 = 0% | Serious* | Not serious | Not serious | Not serious | Not exist # | ⊕⊕⊕◯  Moderate |
| Need for urgent dialysis | 5 | RCT | 580 | 581 | 0.54, [0.17, 1.71] | P = 0.84  I2 = 0% | Serious* | Not serious | Not serious | Not serious | Not exist # | ⊕⊕⊕◯  Moderate |
| Conversion to emergency PCI | 4 | RCT | 507 | 505 | 0.68, [0.3, 1.51] | P = 0.59  I2 = 0% | Serious* | Not serious | Not serious | Not serious | Not exist # | ⊕⊕⊕◯  Moderate |

**Table S2**: summary of GRADE approach

* Due to high or unclear risk of bias domains across included studies

** Due to unexplained heterogeneity between studies.

# We could not assess the publication bias using funnel-plot-based methods because they are inaccurate for fewer than 10 studies reporting the same outcome

**Table S3:** Meta regression analysis for primary outcome (CIN)

| Predictor | Estimate | SE | p |
| --- | --- | --- | --- |
| S.Cr (Base) | 0.7413 | 0.6758 | 0.272 |
| eGFR (Base) | -0.0147 | 0.0131 | 0.264 |
| Contrast volume | -0.0095 | 0.0072 | 0.1839 |
| Age | 0.0256 | 0.0467 | 0.58 |
| BMI | -0.0624 | 0.0959 | 0.515 |

**Table S4:** Meta regression analysis for predicting serum creatinine

| Predictor | Estimate | SE | p |
| --- | --- | --- | --- |
| Age | -0.3634 | 0.318 | 0.2532 |
| BMI | 0.1828 | 1.11 | 0.87 |
| Contrast volume | -0.0526 | 0.077 | 0.495 |
| eGFR (Base) | -0.0564 | 0.1121 | 0.6147 |
| S.Cr (Base) | 3.6281 | 3.6867 | 0.3251 |

**Table S5:** Meta regression analysis for predicting eGFR

| Predictor | | Estimate | | SE | | p | |
| --- | --- | --- | --- | --- | --- | --- | --- |
| Age |  | 0.1194 |  | 0.4 |  | 0.7653 |  |
| BMI |  | 0.8685 |  | 1.12 |  | 0.4368 |  |
| S.Cr (Base) |  | -3.7378 |  | 3.46 |  | 0.28 |  |
| eGFR (Base) |  | 0.1112 |  | 0.1118 |  | 0.3198 |  |
| Contrast volume |  | 0.051 |  | 0.139 |  | 0.72 |  |
